# Seasonal Diet Changes and Trophic Links of Cold-Water Fish (*Coregonus albula*) within a Northern Lake Ecosystem

**DOI:** 10.3390/ani14030394

**Published:** 2024-01-25

**Authors:** Nadezhda A. Berezina, Piotr M. Terentjev, Elena M. Zubova, Sergey M. Tsurikov, Alexey A. Maximov, Andrey N. Sharov

**Affiliations:** 1 Zoological Institute, Russian Academy of Sciences, 199034 St. Petersburg, Russia; 2 Subdivision of the Federal Research Center “Kola Science Center”, Institute of North Industrial Ecology Problems, 184209 Apatity, Russia; p.terentjev@ksc.ru (P.M.T.);; 3 A.N. Severtsov Institute of Ecology and Evolution, Russian Academy of Sciences, 119071 Moscow, Russia; smtsurikov@rambler.ru; 4 Papanin Institute for Biology of Inland Waters, Russian Academy of Sciences, 152742 Borok, Russia; sharov_an@mail.ru

**Keywords:** arctic lakes, cold-water fish, feeding ecology, food web, isotope analysis, seasonality

## Abstract

**Simple Summary:**

Climate change in the high latitudes may endanger cold-water fish that have adapted to the low water temperatures and long winters. Northern oligotrophic lakes, with their naturally sparse food basis for fish, may be especially vulnerable to climate change. However, many aspects of northern fish food ecology have received little attention. We evaluated the dietary habits, trophic positions, and food web interactions of the European vendace, which is regarded as one of the most vulnerable commercial fishes due to modern environmental changes. Using two analyses, fish stomach content and stable isotopes (carbon and nitrogen), we tracked the flow of nutrients through the food web of an example lake (at the White Sea basin) and determined the vendace’s main food sources. In spite of clear seasonal differences in the food preferences of vendace, we determined planktonic copepods to be its key energy (carbon) source. The vendace has also adapted to consume its own embryos during the winter, increasing its trophic position in the lake food web. These findings contribute to a better understanding of the vendace’s feeding habits, its ability to adapt to low-trophic supplies, and the effects of environmental change.

**Abstract:**

The seasonal feeding patterns of the cold-adapted fish, *Coregonus albula*, are poorly studied in high-latitude lakes but could provide insight for predicting the effects of global warming. We examined vendace’s diet composition, traced the carbon and nitrogen isotope ratios from producers to consumers in the food web, and estimated vendace’s trophic position in a subarctic lake (the White Sea basin). Results showed the vendace to be a typical euryphagous fish, but clear seasonal differences were found in the relative importance of plankton and benthos in the diet. The vendace consumed primarily benthic amphipods in the summer, planktonic cladocerans in the autumn, and copepods in the winter–spring (under ice); larvae of aquatic insects were the second-most important food items throughout the year. Because of the substantial proportion of fish embryos in its diet, the vendace had a trophic position similar to that of a predatory fish (perch). The Bayesian food source-mixing model revealed that the majority of vendace energy derives from planktonic copepods. The dominant *Cyclops* had the lowest carbon isotope values, suggesting a carbon-depleted diet typical for methanotrophic bacteria, as its probable food source was in a lake under ice. Understanding the feeding patterns of vendace provides information to better predict the potential biotic effects of environmental change on lake ecosystems.

## 1. Introduction

The impact of climate change on water resources, urban environments, agriculture, human health, and the economy has received attention in many studies [1,2,3,4]. As it strengthens over time, global climate change will become a more powerful stressor for fish living in natural ecosystems [3]. In nonurbanized areas with an absence of pollution, such as the Arctic region, pristine lakes still contain populations of cold-adapted fish species that are vulnerable to global warming [4]. The climate influences fish through a wide range of processes, both directly through metabolic and reproductive processes and indirectly through phenology, prey, predators, and competitors ([5] and references herein). The importance of climate change in the dynamics and exploitation of freshwater fish populations has long been recognized [5]. The European vendace, *Coregonus albula* (Linnaeus, 1758), has a native distribution area in the cold waters (fresh and brackish) of the basins of the Baltic, White, and Barents seas [6]. The eastern distribution border of the vendace reaches the Pechora river basin, when it is replaced by Siberian forms of small-sized whitefish (the least cisco) [7]. The vendace has been introduced into northern lake systems, including the Inari-Pasvik watercourse [8,9]. It is an essential commercial species and an object of fisheries in some northern and eastern countries [9,10].

A clear, increasing trend toward eurythermal species with the warming in lakes has been found, while cold-water species such as coregonids have responded negatively to increasing temperatures [4]. Mild winters leading to early ice-off dates have been detrimental to vendace recruitment in many lakes [4]. Although the rapid warming of water after the hatching of larvae in spring generally enhances vendace survival, late summer temperatures in the southernmost vendace lakes have occasionally risen so high as to increase adult mortality and decrease the size of the vendace population [4,11]. On the contrary, warming has apparently favored the spread of vendace further north and expansion into the subarctic region [12,13]. The date of the ice break and subsequent temperature development, together with resource limitations due to density dependence or competition between age classes, are important factors influencing vendace year–class variance. Earlier ice-breaking triggers make the vendace larvae highly vulnerable to predation at their shorter length (8–15 mm total), with a shorter early larval period resulting in higher larval mortality [12]. An additional indirect climate effect is that the predation pressure from perch on young vendace has actually increased with warmer summers in some lakes, mainly in the north [4].

The diet composition of vendace in northern and other regions has been investigated mainly during the warm season [14]. The vendace has previously been thought to be planktivorous, feeding mostly on zooplankton, ichthyoplankton, and phytoplankton [14,15]. The vendace shows great flexibility and a varied food spectrum [16]. There have recently been numerous reports of vendace feeding on benthic invertebrates, surface insects, and even fish [16,17,18,19]. Some researchers [20] have shown that the composition of vendace food is related to its location in lakes, whether in pelagic or on the bottom (such as in Lake Stekhlin). Vendace captured near the bottom were found to have diets that contained up to 50% biomass from benthic species, but pelagic vendace were mainly planktivorous, preferring planktonic cladocerans and avoiding cyclopoid and calanoid copepods [20]. The mass development of cladocerans, the primary food source for vendace, is restricted to the surface layers. As a result, during the summer, the vendace is planktivorous and occupies the upper 5–10 m layer; during the prespawning period, October–November, it falls to bottom habitats, where the food composition can vary.

Vendace spawn in autumn, and the embryos develop on the bottom over the winter [21,22]. Larvae hatch somewhat before the spring ice-out, and despite differences in first-year growth depending on lakes and years, all one-summer-old juveniles normally recruit to the seine net fishery in autumn. Those who survive their first winter and second summer normally mature and spawn for the first time in their second autumn, and depending on the lake, some may live to spawn again in subsequent years [21,23]. Euryphagy is the most common type of fish feeding in boreal and arctic zones, where food supplies are unstable and vary seasonally in the availability and abundance of food organisms [16,24].

Because freshwater food web research has been focused on temperate lakes, with just a few studies undertaken at high latitudes in Europe [25,26], there is a lack of understanding of feeding ecology in northern vendace populations. Forecasting how these cold-water fish and the lake food chain as a whole will react to current global changes is impossible due to a lack of information regarding how these fish trophic links work. Previously, freshwater lake food webs were characterized by high levels of omnivory by predatory fish [27,28]. We may assume that vendace’s potential to obtain energy from several trophic levels would not only help it survive in the low productivity conditions of northern lakes but would also increase its ability to adapt to global changes.

Stable isotopes of nitrogen and carbon are frequently used to determine the mix of diet sources for consumers. The evaluation of these isotope values in fish and their prey has become a standard technique in food web research, with wide application to aquatic ecosystems [29]. It is a useful tool for investigating trophic interactions and the dynamics of organic matter in food chains because the difference in the ratio of carbon and nitrogen isotopes, Δ^13^C and Δ^15^N, between the consumer and its diet leads to their natural enrichment during the transition from the first to the next trophic level due to metabolic activities [30]. Stable isotopes of nitrogen are highly helpful for determining the trophic position of consumers in aquatic ecosystems.

This study aimed to address the following important question: what are the key energy sources for vendace *C. albula,* and how can the trophic interactions between this fish species and other lake food web members change due to climate variability? To understand the key energy sources of the vendace and the trophic interactions between it and other members of the lake food web, we evaluated vendace diet composition and investigated the ratio of stable isotopes of carbon (^13^C/^12^C, expressed as δ^13^C) and nitrogen (^15^N/^14^N, expressed as δ^15^N) in the tissues of vendace, other fish, and fish food items. These characteristics were studied in the adult part of the vendace population in a small subarctic lake in northern Karelia (northwestern Russia) throughout the year (winter, spring, summer, and autumn). To examine diet composition, the contents and contributions of the main food components in the vendace stomach were investigated. We expected that winter food scarcity and seasonal changes in prey composition and abundance would influence vendace feeding patterns.

## 2. Materials and Methods

### 2.1. Lake Ecosystem Description

**Figure 1:**
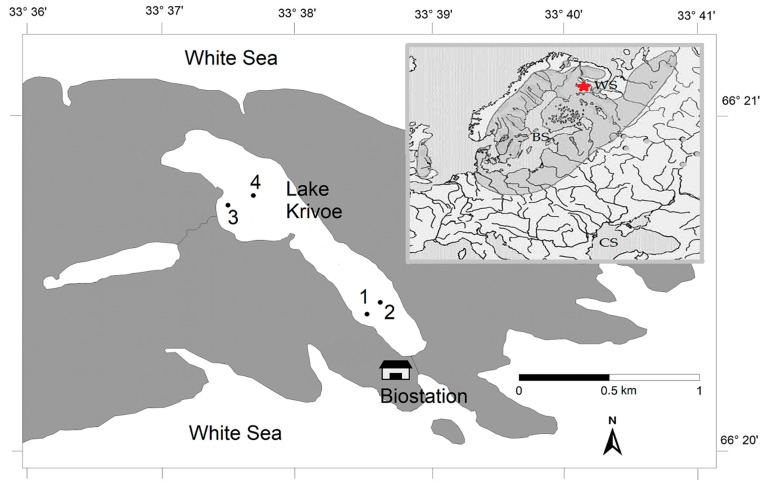
Map showing the location of fish sampling stations (St. 1–4) in Lake Krivoe. The insert map shows the distribution area of vendace. The asterisk indicates the location of the study lake. BS—the Baltic Sea; WS—the White Sea; CS—the Caspian Sea.

The small subarctic lake Krivoe (Kartesh, 66°3435 N, 33°6375 E) is located in North Karelia on the shore of the Kandalaksha Bay of the White Sea (Figure 1 and Figure S1), 30 km south of the Arctic Circle. This area belongs to the Atlantic–Arctic climatic zone. The lake is characterized by cold water and low levels of chlorophyll-A (0.2–3.0 μg/L) and nutrients in the water [31]. From November (or the beginning of December in some years) to the middle of May, it is covered with ice (this is the subice phase for the ecosystem of the lake). From late March to early May (the subice lake phase), hypoxic and anoxic conditions (from 0 to 2.5 mg O_2_/L) form in the bottom layers in the deep-water areas of the lake. After ice melting at the beginning of the open water period (June), the lake has the highest water transparency (4.3–4.7 m).

The structure of the lake’s ecosystem has been studied for more than 50 years, starting in the late 1960s. As a rule, these were studies during the period of open water, and only in the 2019–2021 period were subsurface studies carried out not only in the summer–autumn period but also in the winter–spring period (that is, under the ice). Three fish species—Eurasian perch *Perca fluviatilis*, European vendace *Coregonus albula*, and nine-spined stickleback *Pungitius pungitius*—were recorded in the lake [31]. The phytoplankton consisted mainly of the cyanobacteria *Dolichospermum lemmermannii* and *Coelosphaerium kuetzingianum*, the green colonial microalga *Botryococcus braunii*, and several cryptomonad species (*Komma caudata* and *Cryptomonas* spp.). In addition, other cyanobacteria, such as *Microcystis pulverea* and *Gloeocapsa* spp., were frequent species [32]. The cyanobacteria formed periodically intensive blooms during June and September. The periphyton was mostly composed of the filamentous chlorophyte *Ulothrix zonata* and the benthic cyanobacteria *Scytonema subtile*, *Tolypothrix* sp. and *Phormidium* sp. [33].

Zooplankton biomass is dominated by the two most common groups of planktonic crustaceans, including cladocerans and copepods. The most abundant species were two species of cladocerans, *Bosmina longirostris* and *Sida crystallina*, and the cyclopoid copepod, *Cyclops scutifer* [32,34].

Amphipod crustaceans (*Gammarus lacustris, Monoporeia affinis*, and *Gammaracanthus loricatus*), bivalve mollusks (*Sphaerium corneum* and *Pisidium crassum*), and gastropod mollusks (*Lymnaea stagnalis* and *Planorbis* sp.) were the most abundant benthic organisms in this lake. Ephemeropterans (*Ephemera vulgata* and *Caenis horaria*), trichopterans (*Phryganea bipunctata* and *Limnephilus* sp.), and megalopteran *Sialis flavilatera* contributed notably to the benthic biomass of the lake. *Sergentia coracina*, *Procladius choreus*, and species of the genera *Ablabesmyia* were the most frequent taxa of chironomid dipterans [34].

### 2.2. Sampling and Laboratory Procedures

The studies of fish and their food base (planktonic and benthic origin) were carried out in June, July, September, late October–November (2019, 2020), February, and April (2020 and 2021). Fish were caught at four stations located in the deep-water (St. 1 and 2) and shallow-water (St. 3 and 4) parts of the lake. A total of 107 vendace individuals of vendace were collected to process stomach content analysis. Additionally, in 2019–2020, we collected planktonic and benthic organisms at the same sites using a planktonic net and sediment grab to analyze stable carbon and nitrogen isotopes in their tissues. The muscles from the dorsal body parts of fish (vendace, perch, and stickleback) were taken at each sampling date. We used six individuals per species and date.

In summer and autumn (open water), fish were caught with standard sets of fixed monofilament gillnets. Nets with a length of 30 m, a height of 1.5 m, and a mesh size of 15–18 mm and 24 mm were installed near sampling stations (St. 1–4, Figure 1) at depths from 3 to 28 m. In winter, we collected fish under the ice at the same locations, using a special machine to establish fish gillnets under ice (Figure S2). The mass of the fish was determined within an accuracy of 1 g, and the total length (TL) and standard length (SL) were measured within an accuracy of 1 mm [35]. After each fish was measured and weighed, its stomach was taken from the body cavity, weighed, and analyzed immediately. In cases where deep identification of plants or animals from the stomach was necessary (for example, with a higher magnification of the microscope), they were fixed with 4% buffered formaldehyde (standard for the preservation of aquatic organisms).

### 2.3. Stomach Content Analysis

The total stomach fullness was determined visually on a percentage scale ranging from empty (0%) to full (100%). The stomach contents of fish were examined under an MBS-10 binocular stereomicroscope in a Petri dish. All food components were recognized (potentially to the species level) and classified into taxonomic groups (Cladocera, Copepoda, Amphipoda, Trichoptera, Ephemeroptera, Bivalvia, and so on). Representatives of Amphipoda (*Gammarus*, *Monoporeia*, and *Gammaracanthus*) were considered separately when in analyzing the trophic links in the lake.

The number of specimens (N; specimens per fish stomach or food bolus) was recorded. The mass of the components (mg/stomach) was calculated within an accuracy of 0.01 mg with a Pioneer PX124 electronic balance (OHAUS, Parsippany, NJ, USA).

Using formula (1), the relative importance index (RI) of each food component was calculated as the sum of the component’s percentage (%) contribution to three variables: total number (N), total mass (M) of the food bolus in the fish stomach, and frequency of occurrence (FO) of the component in the total number of analyzed fish. This index is widely used [36,37].

RI = 100 × (%FO + %N + %W)/∑1…n (%FO + %N + %W)(1)

### 2.4. Stable Isotope Analysis

Seston and zooplankton were separated from water samples via filtration through a sieve net (mesh size 300 μm) and placed on filters. Plant, invertebrate, and fish tissues were cleaned in deionized water. Crustaceans were collected without separating the carapaces, giving preference to freshly molted individuals. According to the previously identified strong positive relationship between the carbon isotopic compositions of exoskeletons and their bodies in freshwater planktonic crustaceans [38] and in marine and land arthropods [39,40], the deviations of which are 1–2%, we found it possible to analyze cladocerans, copepods, and amphipods with their whole bodies.

All samples were transferred to a beaker with 10% HCl for five minutes to remove carbonates and then rinsed carefully with deionized water. The samples were dried for at least 48 h in a thermostatically controlled oven at a temperature of 60 °C. The samples were kept at −20 °C prior to stable isotope analysis. 

The analysis of in-organism tissues was performed using an isotope mass spectrometer (Isoprime visION) and an elemental analyzer (Vario ISOTOPE Select, Elementar, Langenselbold, Germany) at the Joint Usage Centre (Instrumental Methods in Ecology), A.N. Severtsov Institute of Ecology and Evolution, Russian Academy of Sciences, Moscow, Russia. Sample portions of all tissues (from 50 to 1250 μg, depending on the nature of the material) were wrapped in tin foil and weighed on a Mettler Toledo MX5 balance (Mettler-Toledo, Columbus, OH, USA). The nitrogen and carbon isotopic compositions are given in thousands of deviations (δ) from the international standard (atmospheric nitrogen and VPDB, respectively):

δX (‰) = [(Rsample/Rstandard − 1)] × 1000(2)

where X is the element (nitrogen or carbon), and R is the molar ratio of heavy and light isotopes. The carbon and nitrogen isotope ratios are expressed in delta (δ) notation relative to Vienna PeeDee Belemnite limestone for δ^13^C and atmospheric nitrogen for δ^15^N. Certified batches of casein and alfalfa powder (Elemental Microanalysis Ltd., Okehampton, UK) were used as working laboratory standards. The standard deviations of δ^15^N and δ^13^C values in laboratory standards were <0.15‰. Additionally, the carbon and nitrogen contents (%C, %N) and the mass ratio of these elements (C:N) were determined in each sample.

### 2.5. Calculations

The C:N ratio was used as a proxy for the animal lipid content. Since lipid extraction may be critical in the analysis of lipid-rich tissue material, the δ^13^C values for consumers with C:N > 3.5 were corrected as recommended by [41]. Lipid-adjusted δ^13^C′ values were recalculated using the following formula (3) [41]:

δ^13^C′ = δ^13^C − 3.32 + 0.99 × C:N (3)

The trophic position (TP) of consumers was calculated from the values of δ^15^N in accordance with [30] and the following formula (4):

TPc = (δ^15^Nc − δ^15^Nb) / Δ^15^N + TPb (4)

where δ^15^Nc is the ratio of nitrogen isotopes in consumers (the taxon in question)l Δ^15^N is the trophic enrichment (fractionation) constant; and δ^15^Nb and TPb are the average nitrogen isotope and trophic position of baseline, respectively, with corresponding constants of Δ^15^N = 3.4% and TPb = 2 [30]. Zoobenthos taxonomic groups with the lowest δ^15^N were selected as the baseline for estimating the TPs, which were larvae of chironomids and mayflies (herbivores and detritivores). Their δ^15^N values were used as the base value of first-order consumers (δ^15^Nb). The TP of true herbivores and detritivores ranges from 2 to <2.5. Species with greater δ^15^N isotope signatures than those of true herbivores were classified as omnivorous consumers (TP > 2.5–3). Those with trophic positions greater than 3 or 4 were classified as first- and second-order predators, respectively.

MixSIAR stable isotope mixing models were used to estimate source proportions (prey importance) in vendace diets (R-statistic, CRAN, GitHub, [42]. Based on RI values, the several dominant prey groups appeared significant in the vendace diet, such as planktonic Cladocera and Copepoda, benthic amphipods (*Monoporeia, Gammarus*), aquatic insects (Ephemeroptera), and bivalve mollusks (Sphaeriidae). In addition, the importance of these prey was evaluated for other predators (predatory amphipods and perch).

Mean values, standard deviation (1SD), and 95% confidence intervals (95%CIs) are presented for the dimensional characteristics of fish and the stable isotope values of the food web groups. The relative importance index of prey in the fish diet is presented as a box plot. The first and third quartiles are shown; the line in the middle of the box is the median (50th percentile), and the whiskers indicate the minimum and maximum observed data values. Differences in the studied parameters between seasons and groups were analyzed using the nonparametric analysis of variance. We applied the Kruskal–Wallis test following Mann–Whitney pairwise comparisons or the Friedman test following Wilcoxon pairwise comparisons. Analyses were performed using the software packages STATISTICA and PAST (https://statistica.software.informer.com/; https://past.en.lo4d.com/, both accessed on 30 November 2023).

## 3. Results

Table 1 presents the data on the length and weight of the vendace used in the analysis of stomach content on various occasions in the 2019–2021 period. Maximal-sized vendace were caught in April (total length = 285 mm, wet weight = 223 g, Figure S3).

### 3.1. Stomach Content

**Figure 2:**
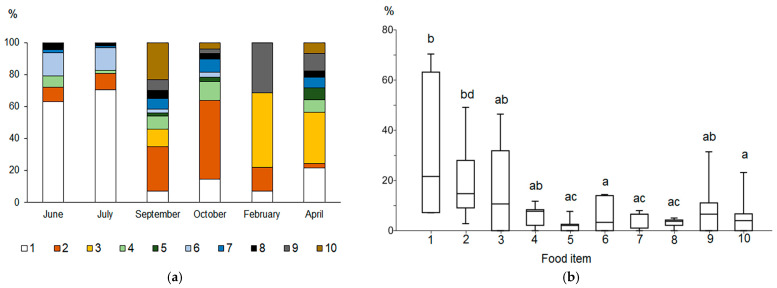
Relative importance index (RI, %) of the main food components of the vendace diet in different periods of the year (**a**) and a box plot of the RI for various food components (**b**). Designations: 1—Amphipoda; 2—Cladocera; 3—Copepoda; 4—Ephemeroptera; 5—Trichoptera; 6—Gastropoda; 7—Bivalvia; 8—Diptera; 9—eggs; 10—detritus. Different letters between pairs (a-b, c-d) show significant differences, and the same letters (a-a, b-b, c-c) between pairs indicate no significant differences (*p* > 0.05) according to Mann–Whitney pairwise comparisons.

The fullness of the vendace stomach varied between dates. It was significantly lower in February (54 ± 34%) than in other months (80–90%, see Table S1, all *p* < 0.05). The food spectrum of vendace, based on analysis of stomach content, contained several dominant groups of benthic and planktonic organisms that played an essential role in the diet (Table S2, Figure 2). Significant differences in frequency of occurrence of all food items were found between months (Wilcoxon pairwise comparison, June and July, *p* = 0.046; July and October, *p* = 0.021; July and April, *p* = 0.013). Significant differences were found between groups (Friedman test, Chi^2^ = 19.91, *p* = 0.011). Wilcoxon pairwise comparison showed differences in the frequency of occurrence between amphipods (species combined) and each of the five items of Gastropoda, Bivalvia, Trichoptera, Diptera, and detritus (all *p* = 0.046).

During the open water period (June, July, September, and October), vendace consumed a variety of species that were predominantly of benthic origin and found in both deep and littoral locations. Among benthic animals, amphipods (*Gammarus* and *Monoporeia*) and ephemeropterans (*Ephemera vulgata*) were the most frequent food items in this period. The contribution of benthic animals decreased in late October when cladoceran crustaceans (mainly *Sida* and, to a lesser degree, *Bosmina*) predominated in the vendace diet. During the ice-covered lake phase (February and April), planktonic copepods (*Macrocyclops*, *Cyclops*, *Eudiaptomus*) were the most frequent items in the diet of vendace (Table S2). In the food of the largest specimen of vendace (24.5 cm, Figure S3), large amphipods *Gammaracanthus* (32–40 mm in body length) from the profundal zone of the lake were found in high numbers. Significant differences in number and abundance contribution between groups were found (Friedman Test, Chi^2^ = 19.2, *p* = 0.02). Furthermore, copepods and fish eggs were distinguished from other food items in order of number (Wilcoxon pairwise comparisons, all *p* = 0.046). The contribution to the total mass of the stomach contents varied significantly between ephemeropterans and amphipods (Wilcoxon pairwise comparisons, *p* = 0.046) as well as between trichopterans and amphipods (*p* = 0.046).

The index of relative importance (RI), which combines the frequency of occurrence and contribution of the food item to the total mass and total number of items in the stomach bolus, revealed significant seasonal differences between food items (Kruskal–Wallis test, Chi*^2^* = 17.34, *p* = 0.04, Mann–Whitney pairwise comparisons). Amphipoda, Cladocera, and Copepoda were the three most common food items in the vendace diet (Figure 2a). Amphipods (*Gammarus* and *Monoporeia*) had the highest relative importance in June (RI = 63.2%) and July (70.4%). Cladocerans were the most important food item in September (28%) and October (49.2%), whereas copepods were the most important in February (46.5%) and April (31.9%). In September, zoogenic detritus contributed 23.2%. In the winter and spring, under ice, fish embryos (vendace) constituted an important part (11.1–31.5%) of the vendace diet. 

The Kruskal–Wallis test found significant differences in relative contributions of various taxa to the vendace diet (Chi^2^ = 17.35; *p* = 0.04). Mann–Whitney pairwise comparisons indicated that amphipods and cladocerans had significantly higher RI values than did aquatic insects (Trichoptera, Diptera), mollusks, and detritus (*p* = 0.005–0.03, see Table S3; Figure 2b).

### 3.2. Stable Isotope Analysis

Table 2 shows the average δ^13^C and δ^15^N values for the dominant trophic web representatives (phytoplankton, periphyton, zooplankton, zoobenthos, and fish) in different seasons. Differences in δ^13^C values between trophic groups were statistically significant for δ^13^C (Kruskal–Wallis test: Chi^2^ = 68.69, *p* < 0.001) as were the differences in δ^15^N values (Chi^2^ = 67.15, *p* < 0.001). Tables S3 and S4 present the Mann–Whitney pairwise comparisons with Bonferroni correction.

**Figure 3:**
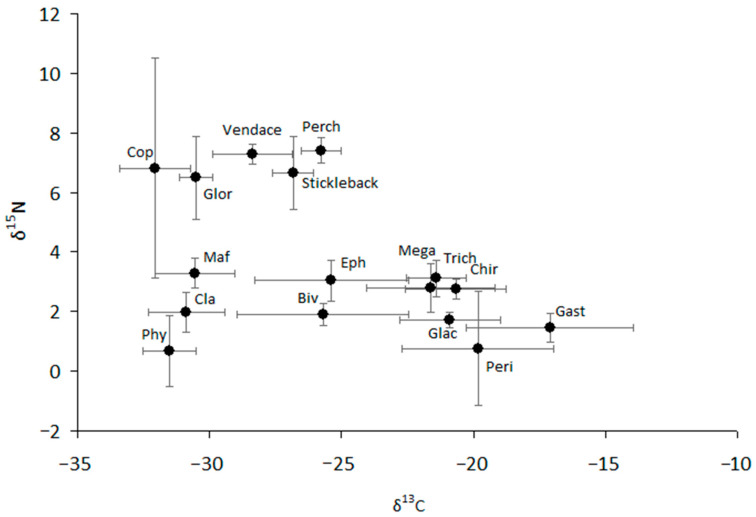
The structure of the food web of the lake. Mean values (based on five separate observations) and 95% confidence intervals are shown. Phy—phytoplankton; Cla—cladocerans; Maf—pontoporeid amphipod *Monoporeia affinis*; Glor—gammarid amphipod *Gammaracanthus loricatus*; Cop—copepod *Cyclops scutifer*; Biv—bivalve mollusk *Sphaerium nitidum*; Eph—ephemeropteran *Ephemera vulgata*; Peri—periphyton (mainly *Ulothrix zonata and *microalgae); Glac—gammarid amphipod *Gammarus lacustris;* Gast—gastropod mollusk *Lymnaea stagnalis*; Chir—chironimid dipteran larvae (mainly *Sergentia coracina*); Trich—trichopteran *Phryganea bipunctata*; Mega—megalopteran *Sialis flavilatera.*

The values of δ^13^C and δ^15^N were highly variable for most studied taxa (Figure 3, Figure S4 and S5). We can separate all consumers into two groups that are different in δ^13^C from each other and closely related to two types of producers, such as phytoplankton and periphyton (Figure 3). Pelagic sources are typically more negative than littoral (bottom) sources, with the difference in δ^13^C between littoral and pelagic sources reaching approximately 7–8‰. Similarly, two carbon flows can be traced from periphyton to littoral consumers (mainly amphipod *Gammarus*, gastropod mollusks, and the larvae of aquatic insects such as chironomids, trichopterans, and megalopterans) and from phytoplankton to zooplankton (Cladocera and Copepoda), benthic amphipod *Monoporeia,* and predaceous benthic amphipod *Gammaracanthus*. Ephemeropteran larvae and sphaeriid mollusks showed very strong variability in carbon composition, the values of which varied over a wide range, from −28.6 to −22.5‰ and from −30.6 to −23.6‰, respectively (Figure 3). The values of δ^13^C of vendace varied from −30.5 to −26.7‰, making them closer to pelagic sources than to littoral sources. Similar to that of vendace, the carbon niches of other fish (stickleback and perch) were intermediate, but their δ^13^C values were closer to periphytic carbon and the littoral food chain than were the δ^13^C values of vendace. As a result, all fish and other consumers (ephemeropterans and bivalve mollusks) with δ^13^C values in an intermediate location could obtain resources from both pelagic and littoral carbon sources in the lake.

**Figure 4:**
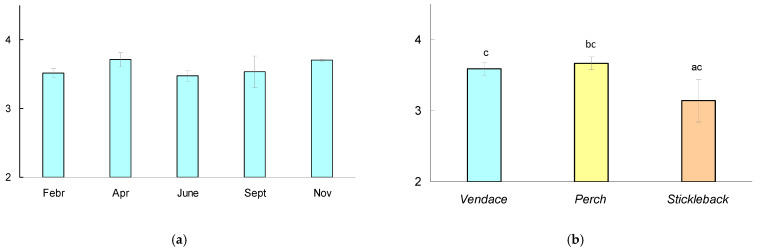
(**a**) Trophic position (TP) of vendace *Coregonus albula* calculated relative to baseline (*Gammarus*, chironomids) during the observation period. The mean values for six fish at each date and the 95% confidence interval are shown. (**b**) TP (mean for five dates and 95% confidence interval) calculated for various species of fish (vendace, perch, and stickleback). The different letters (a-b) show significant differences between pairs in the trophic position of fish at *p* < 0.05, and the same letters (c-c) indicate no significant differences (*p* > 0.05) according to Mann–Whitney pairwise comparisons.

There were no significant differences in the trophic position of vendace between dates; the Mann–Whitney test showed significant differences in trophic position (Figure 4) between perch and stickleback (*p* = 0.027), but there were no differences in the mean trophic position between vendace and perch (*p* = 0.399) or between vendace and stickleback (*p* = 0.06).

Table 3 presents the results of mixed modeling performed for vendace, perch, and a predacious amphipod (*Gammaracanthus*) using mean δ^13^C and δ^15^N values. *Pungitius pungitius* was not included in the model due to its trophic separation from vendace and its preferential consumption of dipteran larvae and pupae as food [43]. Model results indicated close trophic links vendace with three groups: planktonic copepods, benthic amphipods *(Monoporeia* and *Gammarus*), and aquatic insects (*Ephemera*). On average, 68% of vendace’s energy came from the organic matter supplied by copepod crustaceans, followed by amphipods at 13% and aquatic insects at 10%. It was projected that copepod crustaceans might contribute as much as 61% of the food for perch and as much as 90% for the predatory amphipod *Gammaracanthus*, which is the highest predator in the planktonic food chain. The percentage of different amphipod species that vendace consumed was not as much as the that of the amphipod species consumed by perch (Table 3). Cladoceran crustaceans made up a smaller percentage (3–6%) of the diets of all the predator diets in this study, and they contributed more to the diets of predatory amphipods than to fish diets.

## 4. Discussion

We revealed the complex abiotic and biotic interactions in the northern lake environment through examining the trophic structure and feeding patterns of fish (particularly vendace). We found agreement and complementarity between data obtained from combined methodological approaches: (1) stomach contents of fish and (2) carbon and nitrogen stable isotope analysis. Based on the results obtained, vendace turned out to be a typical euryphagous fish and could be classified as an omnivorous-predatory species in the studied lake. The contribution of different prey groups (i.e., relative importance indices of plankton and benthos) to the vendace diet varied throughout the year. During the summer, vendace consumed predominantly benthic species (amphipods). In autumn (September and November), cladocerans made the greatest contribution to their diet, while during the winter–spring period (under-ice lake phase), copepods were the main food items of vendace. The second-most important items in the vendace diet during the year included the larvae of aquatic insects, primarily the larvae of aquatic insects (*Ephemera* and *Phryganea*) and bivalve mollusks (*Sphaerium*). Other research indicates that temporal changes in the diet composition of fish throughout the year are often related to prey availability and energy requirements for reproduction [44]. The vendace has a broader food spectrum in autumn than in other months, probably accumulating energy for subsequent reproduction in late October–November. The trend of stomach fullness, which is a proxy for food demand, showed that the fish were less active in February following the spawning period and at the coldest water temperatures. Simultaneously, it appears that vendace preferred to consume abundant prey of appropriate size throughout the year [16,34]. For example, the favorite prey (amphipods) change their mean size over their life cycle (due to the prevalence of adults or offspring appearance). The dominance in the first half of summer of large mature specimens of *Gammarus* (8–16 mm of body length) in the benthos also determines the high contribution to the feeding of vendace on this prey [34]. In August and September, the size range of *Gammarus*, represented mainly by juveniles (1–4 mm of body length), may limit their use as food by vendace [34].

Vendace spend the open water season in pelagic and littoral environments and the breeding and wintering seasons in deep-bottom habitats. In addition to the high biomass of prey, its location in the lake influences the shift in diet composition [20]. The selection of copepods in this case was related to the peculiarities of the vendace location in the bottom strata, where winter-reproducing copepod species (*Eudiaptomus*, *Cyclops*) are concentrated [34]. However, it seems that the quality of food is also important for fish when they choose food items. The quality of food items is determined by their polyunsaturated fatty acids (PUFAs), including EPA (eicosapentaenoic acid) and DHA (docosahexaenoic acid) content [45]. Among vendace’s food items, amphipods, mayfly larvae, and copepods are considered items of high biochemical quality [46,47,48]. Cladocerans and mollusks have been classified as low-quality objects, as determined by the low content of PUFAs [46,49]. For example, copepods (the main resource in the case study) contain about 20 mg/g of DHA carbon, while cladocerans contain only 1 mg/g of DHA carbon [46]. Apparently, these qualitative properties of potential prey, in addition to their availability, may determine the choice of certain food items by vendace at different periods during the year.

The carbon isotope ratios between a highly specialized consumer and its food, the Δ^13^C values, are thought to be small, ranging from 0.1 to 1‰ [30]. According to our results, only a single species of predatory amphipods (*Gammaracanthus*) can be classified as a highly specialized consumer; the difference in values of δ^13^C between it and its food in terms of Δ^13^C was small (<1‰), on average from 0.1‰ (in comparison with its potential prey, *Monoporeia*) to 0.4‰ (in comparison with its potential prey, Cladocera). Ephemeropterans and bivalve mollusks had the broadest dietary niches, with a wide range of carbon sources (δ^13^C values ranged from −29 to −23‰). This suggests that they have a mixed feeding strategy, including nutrients produced from various producers (algae and bacteria). 

According to the Bayesian mixing model of food sources, vendace could receive the most energy from the organic matter supplied by planktonic crustaceans such as copepods. The large contribution of copepod crustaceans to the vendace diet was confirmed by stomach content analysis, especially during the ice-covered phase (RI = 32–49%). The dominant copepod, *Cyclops*, living in the deepest part of the lake near the bottom had the lowest δ^13^C values obtained in this study (from −34.0 to −30.5‰), i.e., their diet consisted of ^13^C-depleted particles. Biogenic methane is highly depleted in ^13^C due to fractionation during methanogenesis [50]. Thus, methanotrophic bacteria may provide a significant food source for these copepods, especially in winter, and this phenomenon has been observed in many lakes [50,51]. Low δ^13^C values (less than −40‰) have previously been noted for deep-water chironomids [52]. Methanogenesis is the dominant degradation process in anoxic conditions, and the methane produced in lake sediments may subsequently serve as an energy source for methanotrophic bacteria in the water column, representing a link between anoxic (benthic) and oxic (pelagic) communities in the lake [50].

The wide range of obtained δ^15^N values (from −1.5 to 7.9‰) indicates the presence of several trophic levels in the trophic network (from producers to predators and second-order consumers). Four trophic levels were identified in the lake’s food web. The differences in nitrogen isotope composition (Δ^15^N values) between a highly specialized consumer and its food source vary between 3 and 4‰ [37], which did not match ranges found for vendace in our study. The range of Δ^15^N values matched those of highly specialized consumers for four groups: amphipods *Monoporea* (February, April, and November), ephemeropterans *Ephemera* (February to June), trichopterans *Phryganea* (June), and copepods (September). The Δ^15^N values between vendace and other food sources were >4‰. This is explained by the fact that the invertebrates identified in the diet of vendace are mostly omnivorous consumers, using many sources in their diet, and the values of their trophic factors can vary. For example, the value of trophic fractionation (Δ^13^C or Δ^15^N) of aquatic animals, which are detritivores or omnivorous consumers—i.e., use many food sources in their diet—can vary notably and be both higher or lower than the generally accepted values [53].

A large isotopic niche indicates significant differences in diet among individuals [27], while a narrow niche suggests a uniform diet within a population. However, differences in isotopic composition among individuals are not only the result of food sources but also due to differences in isotopic fractionation, which is associated with individual metabolism [54,55]. Changes in growth rate and metabolism may be caused by environmental stressors (for example, hypoxia in wintertime [31]) contributing to variability in δ^15^N values among individuals within species. Additionally, nitrogen supplied from dying bacterial cells or the secondary consumption of nitrogen fixed by cyanoprokaryotes through microbial food webs can contribute to δ^15^N variability [56]. During the open-water period, two to three periodic algae blooms initiated by the cyanobacteria *Dolichospermum* and *Microcystis* were reported from this lake [32], so diazotrophic nitrogen and microbial pathways can be key nitrogen sources for it. All the processes mentioned shift the isotopic compositions, increasing the seasonal variability of the food web’s isotopic signatures.

Identifying the trophic position of fish also helps to reveal the functional role of a species within the trophic web and its specific contribution to energy flow pathways [57,58]. The trophic position of vendace in the studied lake was relatively constant, decreasing only slightly in summer (a period of abundant food availability). Vendace’s trophic position was very similar to that of other predaceous fish, such as perch. Indeed, the perch in this lake is a euryphage with a high proportion of benthic invertebrates in the die (RI 81–83%) and a low proportion of fish (5.6% on average), with only 20% in the largest individuals with a body length > 22 cm [59]. Vendace’s high position in the lake’s food web was also driven by the relatively large contribution of fish embryos (in winter). During the spawning of vendace (late autumn) in other northern lakes (Murmansk region), their own eggs were frequently identified in vendace stomach content [60]. Other fish (for example, stickleback [43]) showed an increase in trophic level if they ate fish eggs. Thus, vendace is an important component of the food web of northern lakes. Being highly adaptive, vendace occupies wide trophic niches, facilitating the circulation of organic matter and ensuring the flow of energy from basal resources to higher levels of the food web. The vendace’s ability to change its diet is likely to provide it with a competitive advantage among generalist fishes following changes in climate and other environmental conditions.

## 5. Conclusions

The feeding habits of adult vendace (*C. albula*) were investigated across all seasons of the year (summer, autumn, winter, and spring) using the example of a small model lake in the subarctic region. This study confirms the opportunistic feeding behavior of vendace and their high adaptability to trophic conditions in the environment. The results of the study revealed strong seasonal variations in the contributions of planktonic and benthic sources to the vendace diet, with benthic and littoral sources dominating in the summer and planktonic sources dominating in the winter. During the year, aquatic larvae of amphibiotic insects, as well as fish eggs during the lake’s ice phase, constituted a constant, although secondary, food source. The trophic position of this “planktivorous” fish was relatively high and similar to that of predatory fish (for example, perch) due to its flexible, omnivorous feeding strategy. In general, the vendace benefits from the lake ecosystem’s food web producers due to its ability to use alternate carbon (methanogenesis) sources in the ice-covered period and nitrogen (diazotrophic bacteria) sources in the open-water period. The use of alternate energy sources by fish may be a common and unique feature of northern lake ecosystems.

We previously assumed that all changes in vendace diet composition would be caused by both resource limitation in an oligotrophic lake (especially in winter) and changes in biotic links (caused by life cycle changes of prey and spatial dislocation of vendace), and these assumptions were partly confirmed. We propose that the composition of the vendace diet may be defined not only by biomass and food availability but also by food quality. In fact, vendace choose the most biochemically valuable and fatty food items (in our case, copepods) as their principal food source. However, more in-depth investigations of vendace’s dietary preferences, taking into account the fatty acid composition of their food, are required to support this hypothesis. Cold-water vendace feeding patterns, examined here for the first time during the under-ice lake phase, provide critical baseline data for monitoring the biotic implications of climate change and other environmental stressors.

## Figures and Tables

**Table 1:** Dimensional characteristics of fish and material volume (n) used for stomach content of vendace by months. Body lengths (TL and SL, mm) and fish mass (WW, g) are given as the min–max, mean and standard deviation, and 95% confidence interval (95% CI). N is the number of analyzed fish.

Time	Parameter	TL	SL	WW
June	Min–Max	148.0–200.0	128.0–184.0	15.0–56.0
	X ± SD	181.6 ± 14.0	161.3 ± 15.6	41.8 ± 10.5
	95%CI	7.1	7.9	5.3
	n	25	25	25
July	Min–Max	156.0–211.0	134.0–185.0	23.0–67.0
	X ± SD	183.0 ± 18.0	158.9 ± 16.4	41.4 ± 14.4
	95%CI	7.2	6.6	5.8
	n	24	24	24
September	Min–Max	128.0–230.0	110.0–205.0	12.0–93.0
	X ± SD	175.1 ± 16.0	153.2 ± 15.5	37.3 ± 12.2
	95%CI	3.9	3.8	3.0
	n	64	64	64
October	Min–Max	120.0–240.0	101.0–213.0	15.0–99.4
	X ± SD	185.5 ± 22.5	160.5 ± 21.9	47.2 ± 17.8
	95%CI	6.7	6.5	5.3
	n	44	44	44
February	Min–Max	169.0–234.0	150.0–217.0	30.0–84.0
	X ± SD	191.4 ± 24.4	172.4 ± 23.6	60.4 ± 20.7
	95%CI	12.0	11.6	10.1
	n	16	16	16
April	Min–Max	110–285	90–264	9–223
	X ± SD	175.0 ± 28.5	152.4 ± 28.7	39.8 ± 38.4
	95%CI	11.0	11.0	14.7
	n	26	26	26

**Table 2:** Values of δ^13^C (‰) and δ^15^N (‰) in tissues of different representatives of the trophic webs of the study lake during different months.

Food Item	Value	δ^13^C′					δ^15^N				
		Feb	Apr	June	Sept	Nov	Feb	Apr	June	Sept	Nov
Phytoplankton	Mean	−31.5	−32.2	−31.7	−29.9	−32.5	1.3	1.4	−1.5	1.1	1.1
	SD	0.3	0.39	0.3	0.0	0.2	0.2	0.2	0.4	0.1	0.5
Periphyton	Mean	−21.5	−18.6	−24.1	−17.0	−18.0	0.9	3.4	1.8	−0.8	−1.5
	SD	0.3	0.6	2.8	0.7	3.9	0.0	0.2	0.7	0.5	0.2
Cladocera	Mean	−30.9	−31.0	−32.3	−29.3	−30.9	2.0	1.4	1.8	2.8	2.0
	SD	0.0	0.2	1.9	0.6	0.0	0.0	0.2	0.8	0.9	0.0
Copepoda	Mean	−30.5	−31.3	−32.7	−34.0	−31.8	10.1	11.1	2.5	3.9	6.5
	SD	0.3	0.0	0.9	3.8	0.2	0.1	0.1	0.6	0.7	0.2
*Monoporeia*	Mean	−28.4	−30.7	−32.8	−30.5	−30.4	3.7	3.6	2.5	3.0	3.6
	SD	0.4	0.7	0.0	0.7	0.8	0.3	0.3	0.0	0.3	0.2
*Gammaracanthus*	Mean	−31.6	−30.2	−30.5	−30.0	−30.4	7.9	6.6	7.9	5.0	5.1
	SD	0.2	0.2	0.1	0.5	0.7	0.1	0.6	0.6	0.8	0.5
*Gammarus*	Mean	−17.9	−20.3	−20.9	−22.4	−22.9	1.5	1.8	2.0	1.4	1.9
	SD	0.6	1.7	0.6	1.2	1.1	0.3	0.5	0.2	0.8	0.6
Bivalvia	Mean	−24.8	−30.6	−23.8	−23.6	−25.7	1.8	2.1	2.3	1.4	1.9
	SD	0.93	2	0.0	1.2	0.0	0.3	0.2	0.0	0.4	0.0
Gastropoda	Mean	−15.7	−14.8	−16.0	−21.9	−17.1	1.1	2.0	1.0	1.7	1.5
	SD	0.0	1.2	0.5	1.8	0.0	0.0	0.7	0.0	0.1	0.0
Ephemeroptera	Mean	−22.5	−22.6	−28.4	−28.6	−24.8	3.6	3.8	3.1	2.0	2.8
	SD	0.9	0.9	0.1	0.0	3.3	0.3	0.5	1.3	0.3	1.0
Trichoptera	Mean	−20.9	−25.8	−20.0	−21.7	−19.7	2.4	2.2	4.2	2.5	2.6
	SD	0.2	0.1	0.0	0.7	0.5	0.3	0.0	0.1	0.8	0.1
Megaloptera	Mean	−20.9	−23.1	−20.9	−20.7	−21.4	2.4	3.9	3.1	3.1	3.1
	SD	0.0	0.1	0.01	0.1	0.0	0.0	0.0	0.0	0.3	0.0
Chironomidae	Mean	−20.0	−19.1	−19.6	−20.5	−24.0	3.1	2.8	3.0	2.5	2.4
	SD	0.9	0.5	0.7	1.6	3.3	0.1	0.4	0.1	0.7	1.0
Stickleback	Mean	−26.2	−27.5	−25.7	−27.4	−27.3	6.2	8.6	7.0	5.2	6.3
	SD	0.32	2.8	0.6	0.8	0.0	0.1	0.6	0.9	0.8	0.0
Vendace	Mean	−30.5	−27.5	−29.4	−27.8	−26.7	6.9	7.6	7.1	7.1	7.7
	SD	0.3	1.0	5.8	2.3	2.9	0.1	0.2	1.3	0.4	0.8
Perch	Mean	−25.9	−26.4	−25.1	−26.6	−24.8	7.3	7.7	7.9	7.1	6.9
	SD	1.0	2.0	1.4	3.3	1.1	0.4	0.3	1.0	0.9	0.6

**Table 3:** Bayesian mixing model predictions performed with MixSIAR package in R in the range of 2.5–97.5% quantiles and the proportion mean ± 1SD values of main food objects (from primary consumers) contributions to the diet of predaceous amphipod *Gammaracanthus loricatus* and two fish species, vendace (*Coregonus albula*) and perch (*Perca fluviatilis).*

	Vendace		*Gammaracanthus*		Perch	
Cladocera	<0.01–0.12	0.03 ± 0.03	<0.01–0.20	0.06 ± 0.05	<0.01–0.46	0.04 ± 0.03
Copepoda	0.61–0.78	0.68 ± 0.04	0.65–0.90	0.76 ± 0.06	0.39–0.61	0.49 ± 0.05
*Monoporeia*	<0.01–0.18	0.05 ± 0.04	<0.01–0.30	0.10 ± 0.08	<0.01–0.20	0.06 ± 0.05
*Gammarus*	<0.01–0.21	0.08 ± 0.05	<0.01–0.21	0.08 ± 0.05	<0.01–0.36	0.17 ± 0.09
Ephemeroptera	<0.01–0.29	0.10 ± 0.07	-	-	<0.01–0.49	0.17 ± 0.13
Bivalvia	<0.01–0.22	0.06 ± 0.05	-	-	<0.01–0.29	0.08 ± 0.07

## Data Availability

The data presented in this study are included in the paper or in the supplementary information file and are available upon request from the corresponding authors.

## Supplementary Materials

The following supporting information can be downloaded at https://www.mdpi.com/article/10.3390/ani14030394/s1L Figure S1: (a) Lake Krivoe in the open-water period (Photo Nadezhda Berezina) and (b) Lake Krivoe in the ice-covered period (Photo Alexey Maximov); Figure S2: Equipment for setting up a gillnet in a lake under ice; Figure S3: View of vendace with minimal and maximal body length from the study lake; Figure S4: Min–max and median values of carbon isotopes in various representatives (1–16) of the food web during the five time periods (February, April, June, September, and late October); Figure S5: Min–max and median values of nitrogen isotopes in various representatives (1–16) of the food web during the five time periods (February, April, June, September, and late October); Table S1: Fullness of stomachs in studied fish; Table S2: Contribution of various food components to the diet of vendace in different periods of the year. FO—frequency of occurrence (%); N—abundance contribution (%); M—mass contribution (%); Table S3: Analysis of RI value variability: Kruskal–Wallis test and Mann–Whitney pairwise comparisons; Table S4: Statistical significance (*p*-value) for differences in δ^13^C between groups in the lake trophic web; Table S5: Statistical significance (*p*-value) for differences in δ^15^N between groups in the lake trophic web.

## References

[1] Kao Y.C., Rogers M.W., Bunnell D.B., Cowx I.G., Qian S.S., Anneville O., Beard T.D., Brinker A., Britton J.R., Chura-Cruz R. (2020). Effects of climate and land-use changes on fish catches across lakes at a global scale. Nat. Commun..

[2] Ficke A.D., Myrick C.A., Hansen L.J. (2007). Potential impacts of global climate change on freshwater fisheries. Rev. Fish. Biol. Fish..

[3] Comte L., Olden J. (2017). Climatic vulnerability of the world’s freshwater and marine fishes. Nat. Clim. Chang..

[4] Jeppesen E., Mehner T., Winfield I.J., Kangur K., Sarvala J., Gerdeaux D., Rask M., Malmquist H.J., Holmgren K., Volta P. (2012). Impacts of climate warming on the long-term dynamics of key fish species in 24 European lakes. Hydrobiologia.

[5] Harrod C., Craig J.F. (2015). Climate change and freshwater fisheries. Freshwater Fisheries Ecology.

[6] Reshetnikov Y.S. (2004). Coregonid fishes in Arctic waters. Ann. Zool. Fenn..

[7] Borovikova E., Makhrov A. (2012). Study of *Coregonus* populations in the zone of intergradation between the vendace and least cisco: The role of the environment in speciation. Princ. Ecol..

[8] Amundsen P.A., Staldvik F.J., Reshetnikov Y.S., Kashulin N., Lukin A., Bøhn T., Sandlund O.T., Popova O.A. (1999). Invasion of vendace *Coregonus albula* in a subarctic watercourse. Biol. Conserv..

[9] Salonen E. (2021). Vendace (*Coregonus albula*) in Lake Inari—What Has Changed in 50 years?. Ann. Zool. Fenn..

[10] Sarvala J., Helminen H., Ventelä A.-M. (2020). Overfishing of a small planktivorous freshwater fish, vendace (*Coregonus albula*), in the boreal lake Pyhäjärvi (SW Finland), and the recovery of the population. Fish. Res..

[11] Mehner T., Emmrich M., Kasprzak P. (2011). Discrete thermal windows cause opposite response of sympatric cold-water fish species to annual temperature variability. Ecosphere.

[12] Helminen H., Sarvala J. (1994). Population regulation of vendace (*Coregonus albula*) in Lake Pyhäjärvi, southwest Finland. J. Fish Biol..

[13] Stewart T.R., Mäkinen M., Goulon C., Guillard J., Marjomäki T.J., Lasne E., Karjalainen J., Stockwel J.D. (2021). Influence of warming temperatures on coregonine embryogenesis within and among species. Hydrobiologia.

[14] Ilmast N.V., Kuchko Y.A. (2023). Zooplankton and feeding of vendace introduced to Lake Vashozero, Lake Onega Basin. Russ. J. Biol. Invasion.

[15] Strandberg U., Hiltunen M., Taipale S.J., Yeung S., Kankaala P. (2018). Planktivorous vendace (*Coregonus albula*) utilise algae-derived fatty acids for biomass increase and lipid deposition. Ecol. Freshw. Fish.

[16] Strelnikova A.P., Berezina N.A. (2021). Diversity of food spectra of vendace in the water bodies of Eurasia. Ecosyst. Transform..

[17] Berezina N.A., Strelnikova A.P., Maximov A.A. (2018). The benthos as the basis of vendace, *Coregonus albula*, and perch, *Perca fluviatilis*, diets in an oligotrophic sub-Arctic lake. Polar Biol..

[18] Scharf J., Krappe M., Koschel R., Waterstraat A. (2008). Feeding of European cisco (*Coregonus albula* and *C. lucinensis*) on the glacial relict crustacean *Mysis relicta* in Lake Breiter Luzin (Germany). Limnologica.

[19] Liso S., Gjelland K.Ø., Reshetnikov Y.S., Amundsen P.A. (2011). A planktivorous specialist turns rapacious: Piscivory in invading vendace *Coregonus albula*. J. Fish Biol..

[20] Schulz M., Kasprzak P., Anwand K., Mehner T. (2003). Diet composition and food preference of vendace (*Coregonus albula* (L.)) in response to seasonal zooplankton succession in Lake Stechlin. Adv. Limnol..

[21] Sarvala J., Rajasilta M., Hangelin C., Hirvonen A., Kiiskilä M., Saarikari V. (1988). Spring abundance, growth and food of 0 + vendace (*Coregonus albula* L.) and white-fish (*C. lavaretus* L. s.l.) in Lake Pyhäjärvi, SW Finland. Finn. Fish. Res..

[22] Lehtonen T.K., Gilljam D., Veneranta L., Keskinen T., Bergenius Nord M. (2023). The ecology and fishery of the vendace (*Coregonus albula*) in the Baltic Sea. J. Fish Biol..

[23] Helminen H., Sarvala J., Hirvonen A. (1990). Growth and food consumption of vendace (*Coregonus albula* (L.)) in Lake Pyhäjärvi, SW Finland: A bioenergetics modeling analysis. Hydrobiologia.

[24] Pavlov D., Kasumyan A. (2002). Feeding Diversity in Fishes: Trophic Classification of Fish. J. Ichthyol..

[25] Kelly B., Amundsen P.-A., Power M. (2022). Trophic niche segregation among native whitefish and invasive vendace in a north Norwegian lake system. Ecol. Freshw. Fish.

[26] Eloranta A.P., Kahilainen K.K., Amundsen P.-A., Knudsen R., Harrod C., Jones R.I. (2015). Lake size and fish diversity determine resource use and trophic position of a top predator in high-latitude lakes. Ecol. Evol..

[27] Thompson R.M., Dunne J., Woodward G. (2012). Freshwater food webs: Towards a more fundamental understanding of biodiversity and community dynamics. Freshw. Biol..

[28] Potapov A.M., Tiunov A.V., Scheu S., Brose U. (2019). Trophic position of consumers and size structure of food webs across aquatic and terrestrial ecosystems. Am. Nat..

[29] Ramírez-García A., Jeppesen E., Moncayo-Estrada R., Mercado-Silva N., Domínguez-Domínguez O. (2023). Diet and trophic structure of the fish community in a small sub-tropical lake in Central Mexico. Water.

[30] Post D.M. (2002). Using stable isotopes to estimate trophic position: Models, methods, and assumptions. Ecology.

[31] Maximov A.A., Berezina N.A., Litvinchuk L.F., Sharov A.N., Maximova O.B., Smirnov V.V., Usov N.V. (2023). Hydrobiological characteristics of small lakes in northern Karelia during the freeze-up period. Proc. Zool. Inst..

[32] Litvinchuk L.F., Sharov A.N., Chernova E.N., Smirnov V.V., Berezina N.A. (2023). Mutual links between microcystins-producing cyanobacteria and plankton community in clear and brown northern lakes. Food Webs.

[33] Gubelit J.I., Nikulina V.N. (2009). Algal community of Krivoye Lake (Northern Karelia) at present time. Biological Resources of the White Sea and Inland Waters of European North. Proceedings of the XXVIII International Conference.

[34] Berezina N.A., Litvinchuk L.F., Maximov A.A. (2021). 2021. Relations between the food spectrum of fishes and the composition of zooplankton and benthos in a subarctic lake. Inl. Water Biol..

[35] Dauvalter V., Terentjev P., Denisov D., Sandimirov S., Koroleva I., Cherepanov A., Kosova A., Kashulin N., Zubova E., Valkova S. (2019). Metody ekologicheskikh issledovanii vodoemov Arktiki (Methods of Ecological Research of Arctic Water Bodies).

[36] Hyslop E.J. (1980). Stomach contents analysis—A review of methods and their application. J. Fish. Biol..

[37] Liao H., Pierce C.L., Larscheid J.G. (2001). Empirical assessment of indices of prey importance in the diets of predacious fish. Trans. Amer. Fish. Soc..

[38] Perga M.E. (2010). Potential of δ13C and δ15N of cladoceran subfossil exoskeletons for paleo-ecological studies. J. Paleolimnol..

[39] Macko S.A., Helleur R., Hartley G., Jackman P. (1989). Diagenesis in organic matter_a study using stable isotopes of individual car-bohydrates. Adv. Org. Geochem..

[40] Webb S.C., Hedges R.E.M., Simpson S.J. (1998). Diet quality influences the δ13C and δ15N of locusts and their biochemical components. J. Exp. Biol..

[41] Post D.M., Layman C.A., Arrington D.A. (2007). Getting to the fat of the matter: Models, methods and assumptions for dealing with lipids in stable isotope analyses. Oecologia.

[42] Stock B.C., Jackson A.L., Ward E.J., Parnell A.C., Phillips D.L., Semmens B.X. (2018). Analyzing mixing systems using a new generation of Bayesian tracer mixing models. PeerJ.

[43] Berezina N.A., Zhgareva N.N., Strelnikova A.P. (2023). Feeding features of the nine-spined stickleback *Pungitius pungitius* (gasterosteidae) in water bodies of the North-West of Russia. J. Ichthyol..

[44] Fanelli E., Principato E., Monfardini E., Da Ros Z., Scarcella G., Santojanni A., Colella S. (2022). Seasonal Trophic Ecology and Diet Shift in the Common Sole *Solea solea* in the Central Adriatic Sea. Animals.

[45] Gladyshev M.I., Sushchik N.N., Kalachova G.S., Makhutova O.N. (2012). Stable Isotope Composition of Fatty Acids in Organisms of Different Trophic Levels in the Yenisei River. PLoS ONE.

[46] Gladyshev M.I., Sushchik N.N., Dubovskaya O.P., Buseva Z.F., Makhutova O.N., Fefilova E.B., Feniova I.Y., Semenchenko V.P., Kolmakova A.A., Kalachova G.S. (2015). Fatty acid composition of Cladocera and Copepoda from lakes of contrasting temperature. Freshw. Biol..

[47] Sushchik N.N., Gladyshev M.I., Moskvichova A.V., Makhutova O.N., Kalachova G.S. (2003). Comparison of fatty acid composition in major lipid classes of the dominant benthic invertebrates of the Yenisei river. Comp. Biochem. Phys. B.

[48] Makhutova O.N., Shulepina S.P., Sharapova T.A., Kolmakova A.A., Glushchenko L.A., Kravchuk E.S., Gladyshev M.I. (2018). Intraspecies variability of fatty acid content and composition of a cosmopolitan benthic invertebrate, *Gammarus lacustris*. Inland Waters.

[49] Makhutova O.N., Shulepina S.P., Sharapova T.A., Dubovskaya O.P., Sushchik N.N., Baturina M.A., Pryanichnikova E.G., Kalachova G.S., Gladyshev M.I. (2016). Content of polyunsaturated fatty acids essential for fish nutrition in zoobenthos species. Freshw. Sci..

[50] Bastviken D., Ejlertsson J., Sundh I., Tranvik L. (2003). Methane as A Source of Carbon And Energy For Lake Pelagic Food Webs. Ecology.

[51] van Duinen G.A., Vermonden K., Bodelier P.L.E., Hendriks A.J., Leuven R.S.E.W., Middelburg J.J., van der Velde G., Verberk W.C.E.P. (2013). Methane as a carbon source for the food web in raised bog pools. Freshw. Sci..

[52] Jones R.I., Carter C.E., Kelly A., Ward S., Kelly D.J., Grey J. (2008). Widespread contribution of methane-cycle bacteria to the diets of lake profundal chironomid larvae. Ecology.

[53] Dionne K., Dufresne F., Nozais C. (2016). Variation in δ^13^C and δ^15^N trophic enrichment factors among *Hyalella azteca* amphipods from different lakes. Hydrobiologia.

[54] Gorokhova E. (2018). Individual growth as a non-dietary determinant of the isotopic niche metrics. Methods Ecol. Evol..

[55] Karlson A.M.L., Reutgard M., Garbaras A., Gorokhova E. (2018). Isotopic niche reflects stress-induced variability in physiological status. R. Soc. Open Sci..

[56] Motwani N.H., Duberg J., Svedén J.B., Gorokhova E. (2018). Grazing on cyanobacteria and transfer of diazotrophic nitrogen to zooplankton in the Baltic Sea. Limnol. Ocean..

[57] Delong M., Thorp J.M., Thons M.S., Mcintosh L. (2011). Trophic niche dimensions of fish communities as a function of historical hydrological conditions in a Plains River. River Syst..

[58] Svanbäck R., Quevedo M., Olsson J., Eklöv P. (2015). Individuals in food webs: The relationships between trophic position, omnivory and among-individual diet variation. Oecologia.

[59] Terentjev P.M., Berezina N.A. (2022). Ecological and morphological characteristics and feeding of perch (*Perca fluviatilus*) in the autumn–winter period in dystrophic and oligotrophic lakes of Northern Karelia (Russia). Inland Water Biol..

[60] Koroleva I.M., Valkova S.A., Vandysh O.I., Denisov D.B., Terentjev P.M., Sandimirov S.S., Dauvalter V.A., Kashulin N.A. (2012). State of the ecosystem of Lake Kovdor and characteristics of the fish part of its population. Proc. Kola Sci. Cent. RAS.

