# Supplementary material for: Seasonal Diet Changes and Trophic Links of Cold-Water Fish (Coregonus albula) within a Northern Lake Ecosystem
Source: Animals (Basel). 2024 Jan 25;14(3):394. doi: 10.3390/ani14030394 (PMC10854978; doi:10.3390/ani14030394)
Supplement: Supplementary file 1 [file animals-14-00394-s001.zip › animals-2789798-supplementary.pdf]

a)

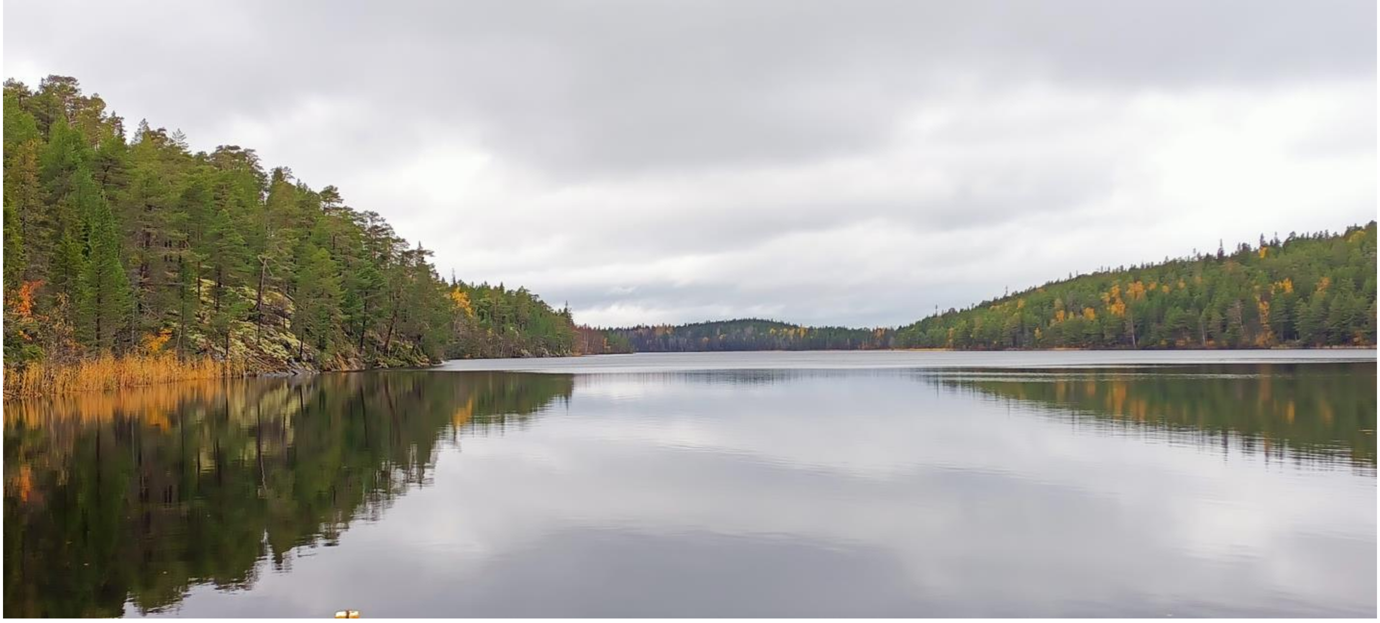

b)

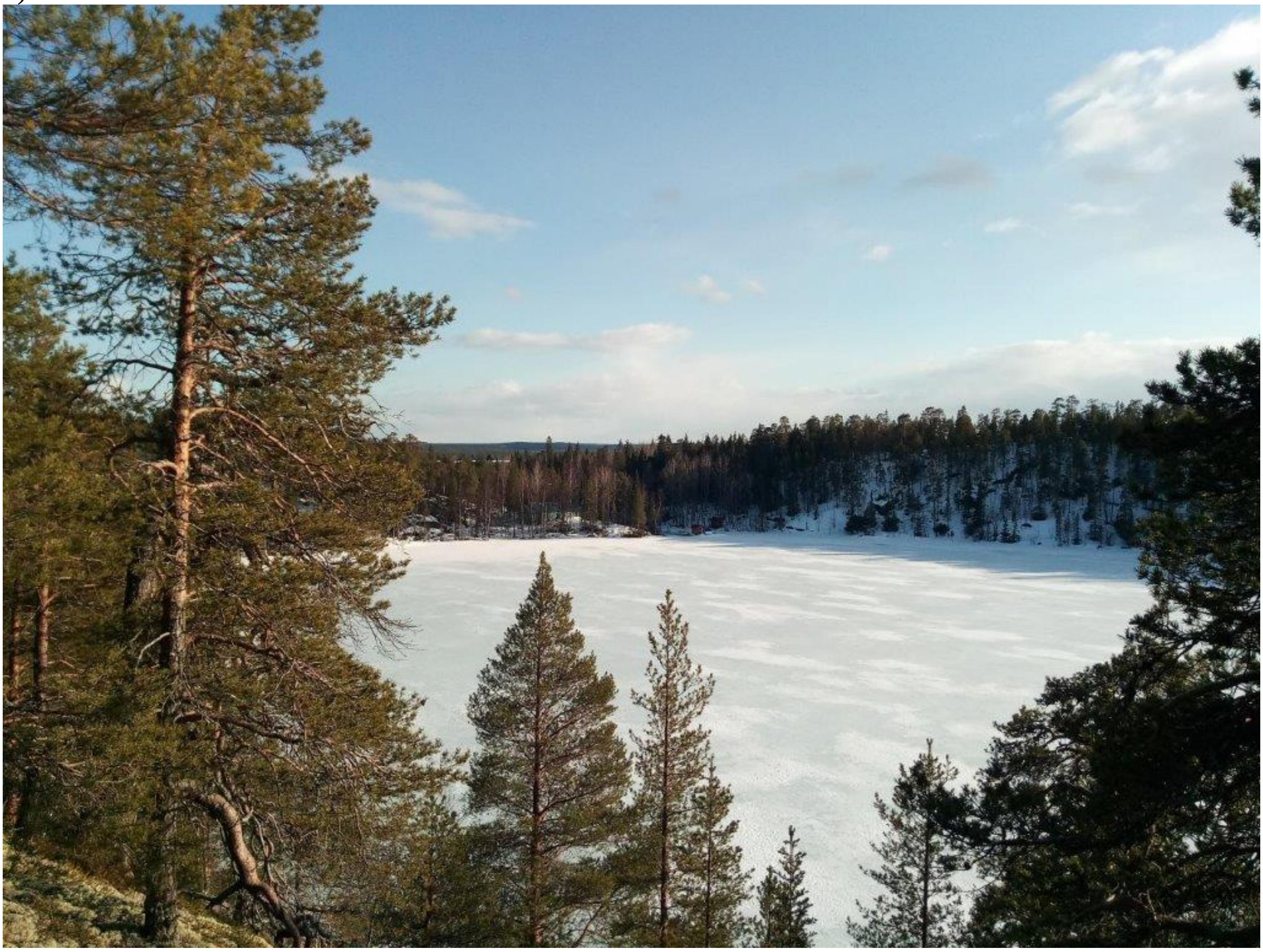

Figure S1. a) Lake Krivoe in open water period (Photo Nadezhda Berezina); b) Lake Krivoe in ice-covered period (Photo Alexey Maximov).

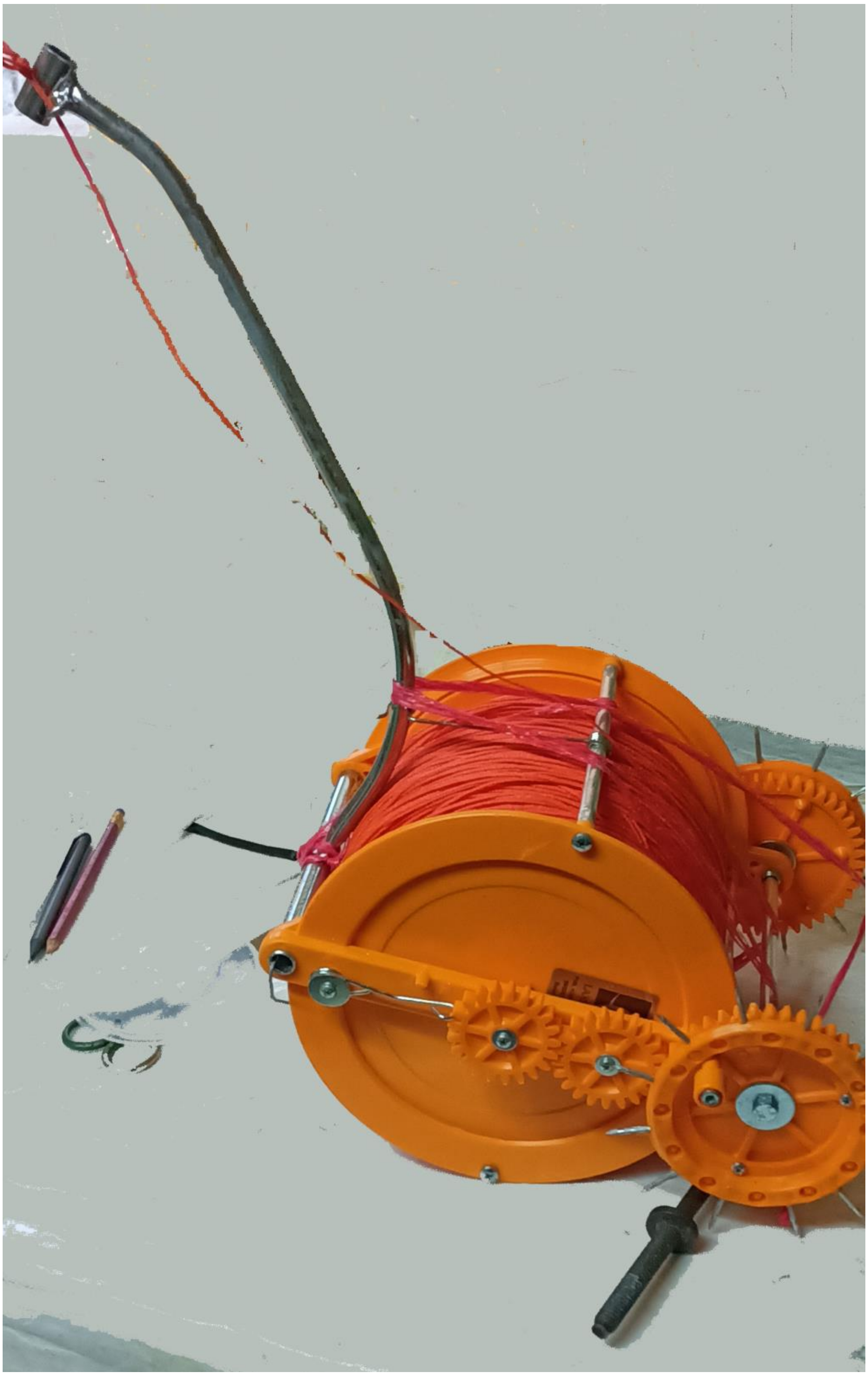

Figure S2. Equipment for setting up a gillnet in a lake under ice.

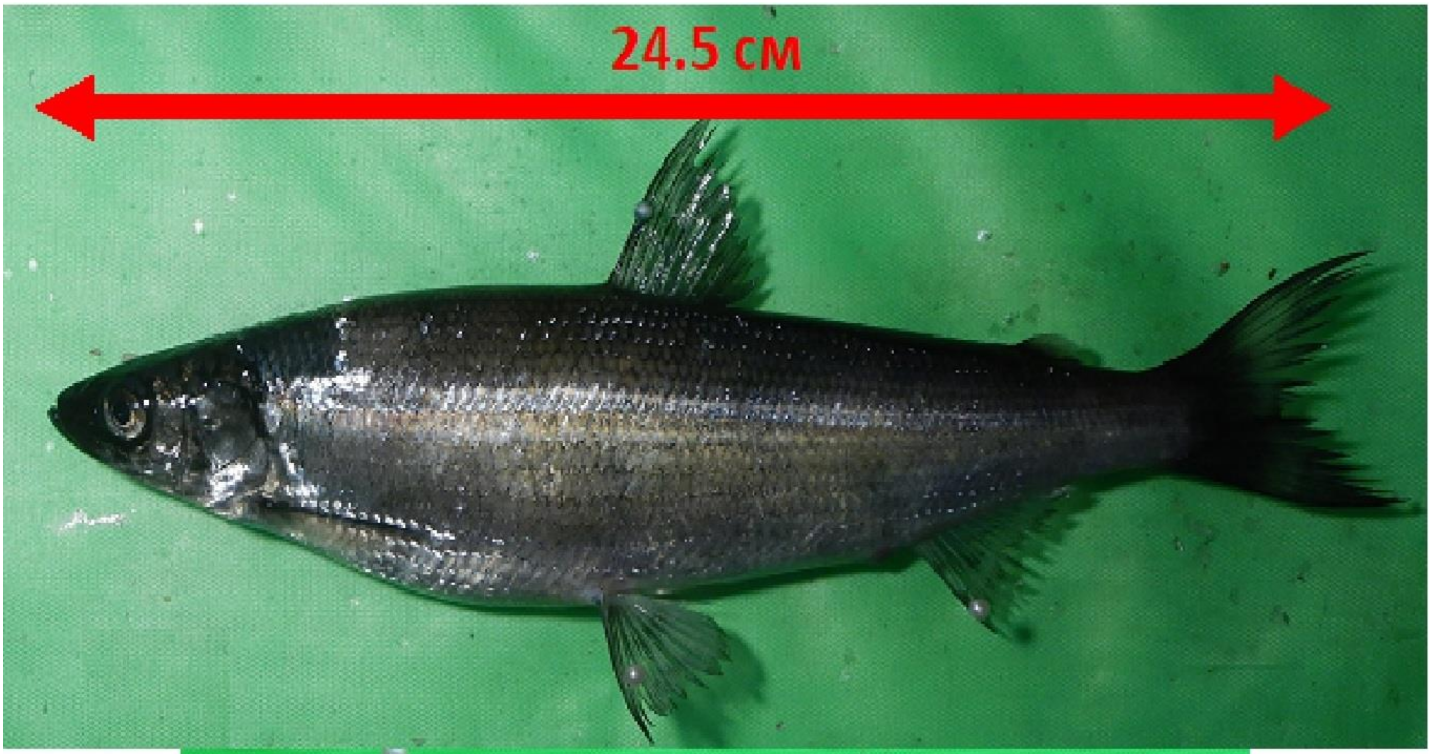

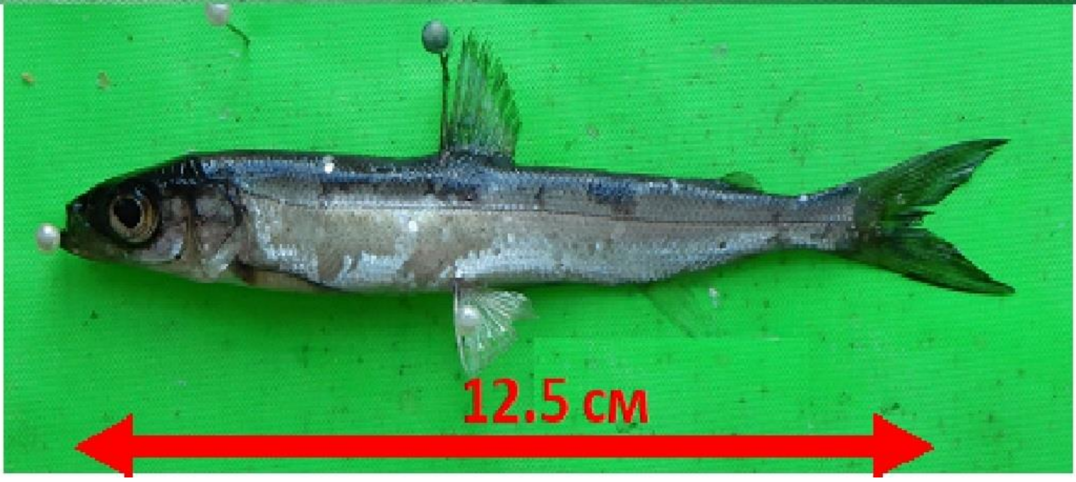

Figure S3. View of vendace with minimal and maximal body length from study lake.

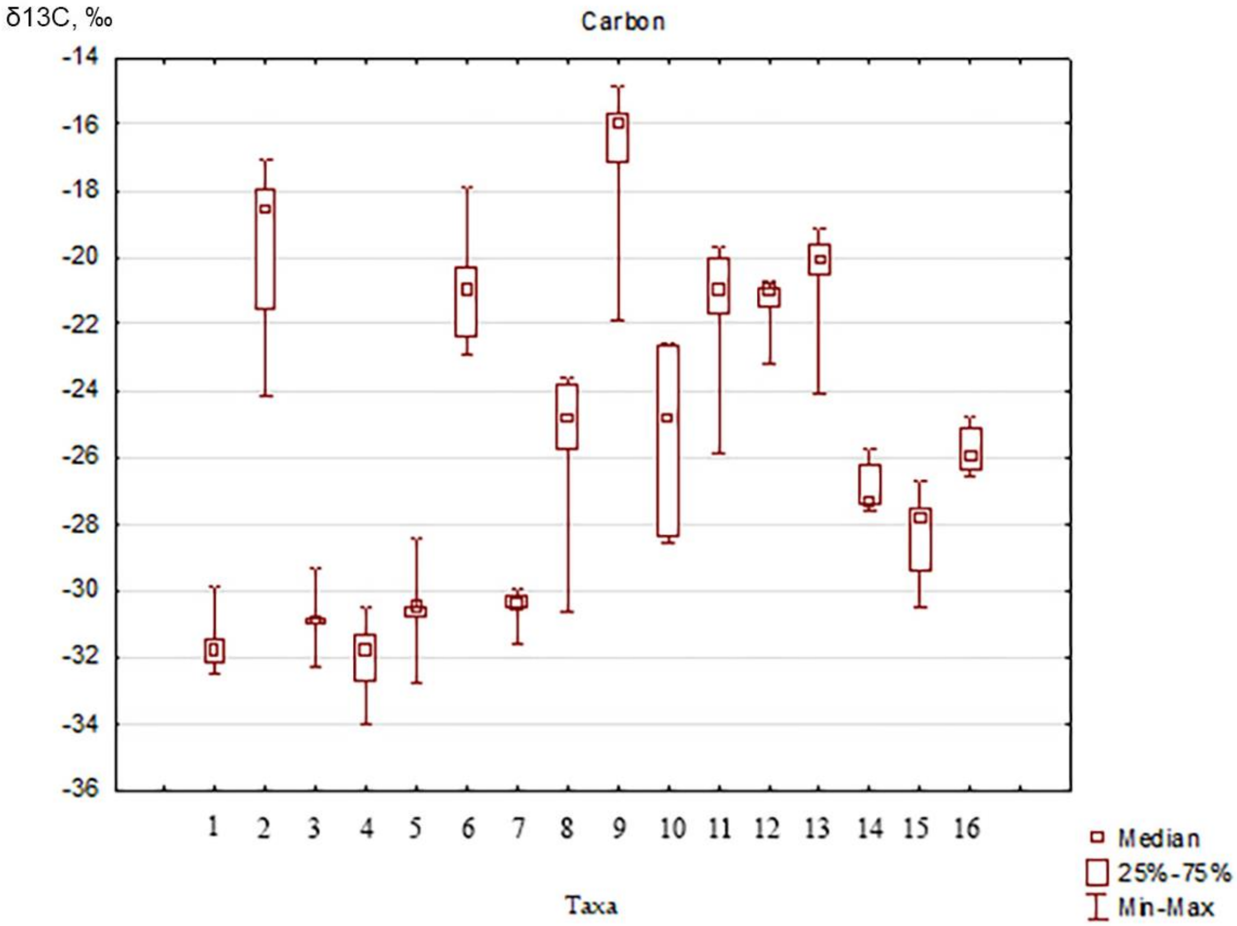

Figure S4. Min-Max and median values of carbon isotopes in various representatives (1-16) of food web during five dates (February, April, June, September and late October). 1. Phytoplankton, 2. Periphyton, 3.Cladocera, 4.Copepoda, 5.Monoporeia, 6.Gammarus, 7.Gammaracanthus, 8.Bivalvia, 9.Gastropoda, 10.Ephemeroptera, 11.Trichoptera, 12.Megaloptera, 13.Chironomidae, 14.Pungitius, 15.Coregonus, 16.Perca

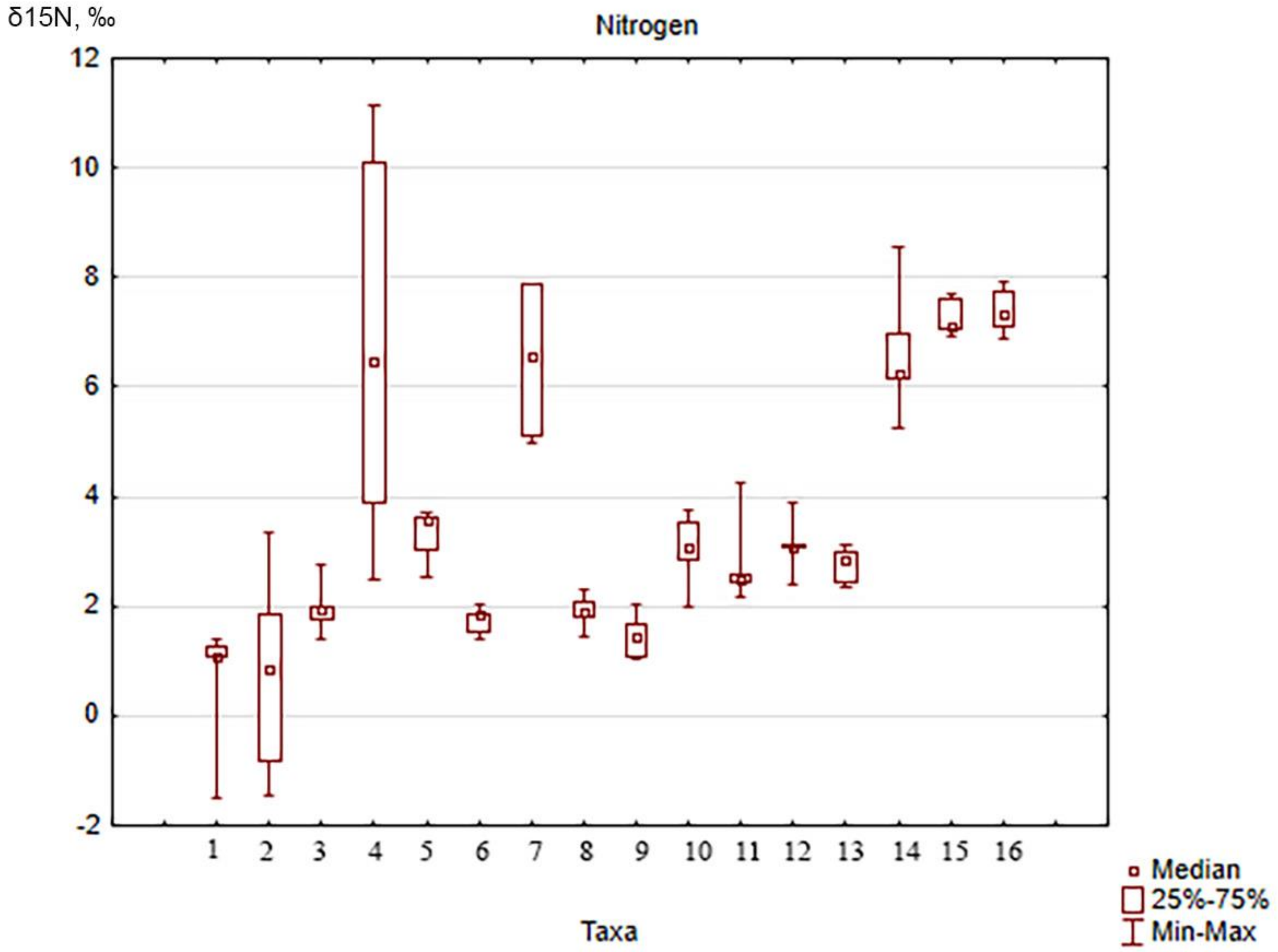

Figure S5. Min-Max and median values of nitrogen isotopes in various representatives (1-16) of food web during five dates (February, April, June, September and late October). 1. Phytoplankton, 2. Periphyton, 3.Cladocera, 4.Copepoda, 5.*Monoporeia*, 6.*Gammarus*, 7.*Gammaracanthus*, 8.Bivalvia, 9.Gastropoda, 10.Ephemeroptera, 11.Trichoptera, 12.Megaloptera, 13.Chironomidae, 14.*Pungitius*, 15.*Coregonus*, 16.*Perca*.

Table S1. Fullness (%) of stomachs in vendace. Mean values, standard deviations (SD) and 95% confidence intervals are presented. Different letters show significant differences between mean values at $p < 0.05$.

Value	June	July	Sept	Oct	Febr	Apr
Mean	88.57[b]	90.00[b]	85.71[b]	80.00[b]	54.29[a]	80.71[b]
SD	17.03	15.19	17.85	31.83	33.68	22.69
95%SI	8.92	7.96	9.35	16.67	17.64	11.89

Table S2. Contribution of various food components to the diet of vendace in different periods of the year. FO is the frequency of occurrence (%), N is the abundance contribution (%), M is the mass contribution (%). "Eph" is Ephemeroptera, "Gast" is Gastropoda, "Biv" is Bivalvia, "Trich" is Trichoptera, "Dipt" is Diptera, "Amp" is Amphipoda, "Cop" is Copepoda, "Clad" is Cladocera, "Eggs" is fish eggs, and "Detr" is detritus.

		Food items									
Month	Index	Eph	Gast	Biv	Trich	Dipt	Amp	Cop	Clad	Eggs	Detr
June	FO	20.0	13.3	6.7	0.0	13.3	73.3	0.0	13.3	0.0	0.0
	N	0.2	9.6	0.2	0.0	0.5	74.6	0.0	14.9	0.0	0.0
	M	2.9	26.2	0.5	0.0	0.8	66.9	0.0	2.7	0.0	0.0
July	FO	5.0	6.0	2.0	0.0	2.0	25.0	0.0	9.0	0.0	0.0
	N	0.3	10.0	0.3	0.0	1.7	73.4	0.0	14.3	0.0	0.0
	M	0.1	18.9	0.4	0.0	1.7	77.0	0.0	1.9	0.0	0.0
September	FO	25.0	3.0	12.5	6.0	8.0	14.0	12.5	19.0	0.0	8.0
	N	0.6	0.1	7.3	0.3	0.3	7.6	20.1	59.7	0.2	3.8
	M	0.2	3.8	0.6	0.1	7.3	0.3	0.3	7.6	20.1	59.7
November	FO	29.5	6.8	15.9	9.1	11.4	18.0	0.0	56.8	6.8	13.6
	N	1.0	1.8	2.4	0.2	0.6	1.8	0.0	90.2	1.3	0.7
	M	12.7	3.8	11.3	0.2	1.6	34.8	0.0	33.9	1.2	0.5
February	FO	0.0	0.0	0.0	0.0	0.0	25.0	62.5	37.5	50.0	0.0
	N	0.0	0.0	0.0	0.0	0.0	0.3	55.9	10.7	33.1	0.0
	M	0.0	0.0	0.0	0.0	0.0	1.8	55.9	7.3	34.9	0.0
April	FO	16.0	0.0	8.0	8.0	12.0	32.0	40.0	8.0	16.0	20.0
	N	0.7	0.0	8.4	0.9	0.7	11.0	57.0	1.6	17.1	2.6
	M	11.3	0.0	7.2	18.8	0.9	35.0	17.8	0.5	6.7	1.7

Table S3. Analysis of RI values variability: Kruskal-Wallis test and Mann-Whitney pairwise comparisons Bonferroni corrected. Kruskal-Wallis test: $Chi^2 = 17.35$ $p < 0.04$.

	Group	1	2	3	4	5	6	7	8	9	10
1	Amphipoda	0.00	0.58	0.23	0.07	0.01	0.03	0.01	0.01	0.07	0.03
2	Cladocera	1.00	0.00	0.47	0.07	0.01	0.09	0.02	0.03	0.17	0.06
3	Copepoda	1.00	1.00	0.00	1.00	0.49	0.93	0.87	0.87	0.93	0.61
4	Ephemeroptera	1.00	1.00	1.00	0.00	0.10	0.94	0.34	0.26	0.81	0.33
5	Trichoptera	0.56	0.35	1.00	1.00	0.00	0.36	0.33	0.25	0.36	0.73
6	Gastropoda	1.00	1.00	1.00	1.00	1.00	0.00	0.94	0.94	0.94	0.93
7	Bivalvia	0.59	0.91	1.00	1.00	1.00	1.00	0.00	0.69	0.75	0.74
8	Diptera	0.23	1.00	1.00	1.00	1.00	1.00	1.00	0.00	0.69	1.00
9	fish eggs	1.00	1.00	1.00	1.00	1.00	1.00	1.00	1.00	0.00	0.68
10	detritus	1.00	1.00	1.00	1.00	1.00	1.00	1.00	1.00	1.00	0.00

Example page after test:

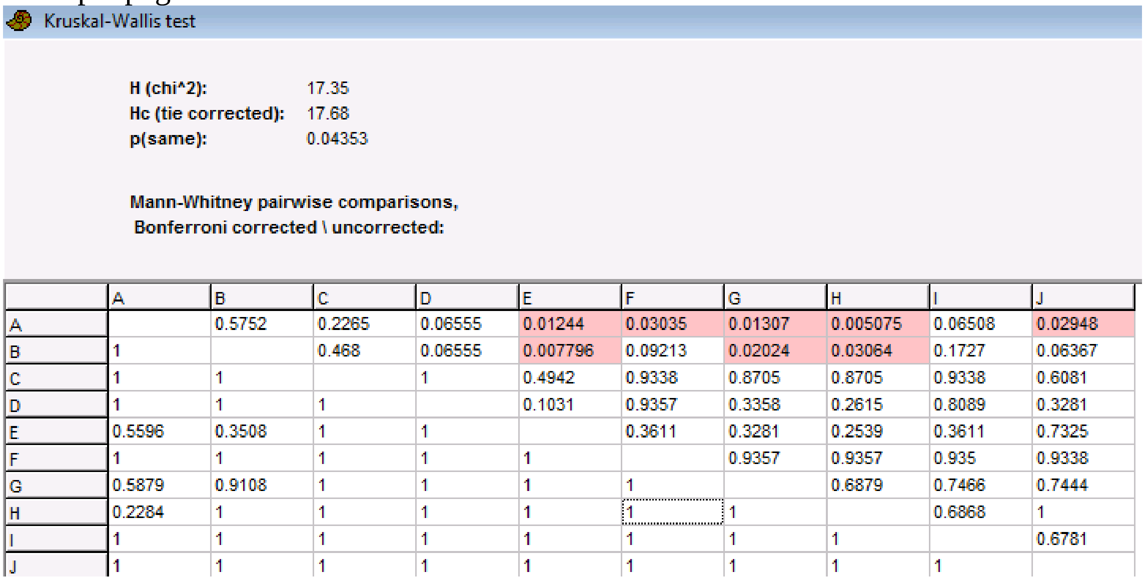

Kruskal-Wallis test

H (chi^2): 17.35
Hc (tie corrected): 17.68
p(same): 0.04353

Mann-Whitney pairwise comparisons,
Bonferroni corrected \ uncorrected:

	A	B	C	D	E	F	G	H	I	J
A		0.5752	0.2265	0.06555	0.01244	0.03035	0.01307	0.005075	0.06508	0.02948
B	1		0.468	0.06555	0.007796	0.09213	0.02024	0.03064	0.1727	0.06367
C	1	1		1	0.4942	0.9338	0.8705	0.8705	0.9338	0.6081
D	1	1	1		0.1031	0.9357	0.3358	0.2615	0.8089	0.3281
E	0.5596	0.3508	1	1		0.3611	0.3281	0.2539	0.3611	0.7325
F	1	1	1	1	1		0.9357	0.9357	0.935	0.9338
G	0.5879	0.9108	1	1	1	1		0.6879	0.7466	0.7444
H	0.2284	1	1	1	1	1	1		0.6868	1
I	1	1	1	1	1	1	1	1		0.6781
J	1	1	1	1	1	1	1	1	1	

Table S4. Statistical significance (*p*-value) for differences in $\delta^{13}C$ between groups of lake trophic web. Kruskal-Wallis test: $Chi^2 = 68.69$, $p < 0.001$. Mann-Whitney pairwise comparisons Bonferroni corrected.

	1	2	3	4	5	6	7	8	9	10	11	12	13	14	15
1.Phytoplankton	0														
2.Periphyton	0.012														
3.Cladocera	0.296	0.012													
4.Copepoda	0.676	0.012	0.210												
5.Monoporeia	0.403	0.012	0.403	0.210											
6.Gammarus	0.012	0.676	0.012	0.012	0.012										
7.Gammaracanth	0.210	0.012	0.403	0.060	0.676	0.012									
8.Bivalvia	0.022	0.037	0.022	0.022	0.060	0.012	0.095								
9.Gastropoda	0.012	0.144	0.012	0.012	0.012	0.060	0.012	0.012							
10.Ephemeropte	0.012	0.037	0.012	0.012	0.022	0.037	0.012	0.676	0.012						
11.Trichoptera	0.012	0.296	0.012	0.012	0.012	1.000	0.012	0.095	0.095	0.060					
12.Megaloptera	0.012	0.531	0.012	0.012	0.012	0.835	0.012	0.012	0.095	0.037	1.000				
13.Chironomida	0.012	0.531	0.012	0.012	0.012	0.676	0.012	0.037	0.095	0.037	0.403	0.144			
14.Pungitius	0.012	0.012	0.012	0.012	0.012	0.012	0.012	0.144	0.012	0.676	0.022	0.012	0.012		
15.Coregonus	0.022	0.012	0.037	0.022	0.075	0.012	0.060	0.144	0.012	0.210	0.012	0.012	0.012	0.095	
16.Perca	0.012	0.012	0.012	0.012	0.012	0.012	0.012	0.403	0.012	0.835	0.037	0.012	0.012	0.144	0.012

Table S5. Statistical significance (*p*-value) for differences in $\delta^{13}C$ between groups of lake trophic web. Kruskal-Wallis test: Chi2 =67.15, $p < 0.001$. Mann-Whitney pairwise comparisons Bonferroni corrected.

	1	2	3	4	5	6	7	8	9	10	11	12	13	14	15
1.Phytoplankton															
2.Periphyton	1.000														
3.Cladocera	0.012	0.296													
4.Copepoda	0.012	0.022	0.022												
5.Monoporeia	0.012	0.037	0.022	0.144											
6.Gammarus	0.016	0.403	0.531	0.012	0.012										
7.Gammaracan	0.012	0.012	0.012	1.000	0.012	0.012									
8.Bivalvia	0.012	0.296	1.000	0.012	0.012	0.403	0.012								
9.Gastropoda	0.403	0.531	0.210	0.012	0.012	0.296	0.012	0.144							
10.Ephemeropte	0.012	0.060	0.028	0.095	0.676	0.022	0.012	0.037	0.022						
11.Trichoptera	0.012	0.095	0.095	0.095	0.210	0.012	0.012	0.022	0.012	0.531					
12.Megaloptera	0.012	0.094	0.021	0.115	0.834	0.012	0.012	0.012	0.012	1.000	0.402				
13.Chironomida	0.012	0.144	0.037	0.060	0.095	0.012	0.012	0.012	0.012	0.463	0.676	0.295			
14.Pungitius	0.012	0.012	0.012	1.000	0.012	0.012	0.834	0.012	0.012	0.012	0.012	0.012	0.012		
15.Coregonus	0.012	0.012	0.012	0.676	0.012	0.012	0.675	0.012	0.012	0.012	0.012	0.012	0.012	0.210	
16.Perca	0.012	0.012	0.012	0.676	0.012	0.012	0.402	0.012	0.012	0.012	0.012	0.012	0.012	0.210	0.531
